# High-Throughput Sequencing Identified Distinct Bipartite and Monopartite Begomovirus Variants Associated with DNA-Satellites from Tomato and Muskmelon Plants in Saudi Arabia

**DOI:** 10.3390/plants12010006

**Published:** 2022-12-20

**Authors:** Khalid A. AlHudaib, Mostafa I. Almaghasla, Sherif M. El-Ganainy, Muhammad Arshad, Nizar Drou, Muhammad N. Sattar

**Affiliations:** 1 Department of Arid Land Agriculture, College of Agricultural and Food Sciences, King Faisal University, P.O. Box 420, Al-Ahsa 31982, Saudi Arabia; 2 Pests and Plant Diseases Unit, College of Agriculture and Food Sciences, King Faisal University, P.O. Box 420, Al-Ahsa 31982, Saudi Arabia; 3 Plant Pathology Research Institute, Agricultural Research Center, Giza 12619, Egypt; 4 Bioinformatics Core, Center for Genomics & Systems Biology, New York University Abu Dhabi, Abu Dhabi P.O. Box 129188, United Arab Emirates; 5 Central Laboratories, King Faisal University, P.O. Box 420, Al-Ahsa 31982, Saudi Arabia

**Keywords:** begomovirus, DNA-satellites, Illumina MiSeq, muskmelon, mixed infection, Saudi Arabia, tomato

## Abstract

The studies on the prevalence and genetic diversity of begomoviruses in Saudi Arabia are minimal. In this study, field-grown symptomatic tomato and muskmelon plants were collected, and initially, begomovirus infection was confirmed by the core coat protein sequences. Four tomato and two muskmelon plants with viral infections were further evaluated for Illumina MiSeq sequencing, and twelve sequences (2.7–2.8 kb) equivalent to the full-length DNA-A or DNA-B components of begomoviruses were obtained along with eight sequences (~1.3–1.4 kb) equivalent to the begomovirus-associated DNA-satellite components. Four begomovirus sequences obtained from tomato plants were variants of tomato yellow leaf curl virus (TYLCV) with nt sequence identities of 95.3–100%. Additionally, two tomato plants showed a mixed infection of TYLCV and cotton leaf curl Gezira virus (CLCuGeV), okra yellow crinkle Cameroon alphasatellite (OYCrCMA), and okra leaf curl Oman betasatellite (OLCuOMB). Meanwhile, from muskmelon plants, two sequences were closely related (99–99.6%) to the tomato leaf curl Palampur virus (ToLCPalV) DNA-A, whereas two other sequences showed 97.9–100% sequence identities to DNA-B of ToLCPalV, respectively. Complete genome sequences of CLCuGeV and associated DNA-satellites were also obtained from these muskmelon plants. The nt sequence identities of the CLCuGeV, OYCrCMA, and OLCuOMB isolates obtained were 98.3–100%, 99.5–100%, and 95.6–99.7% with their respective available variants. The recombination was only detected in TYLCV and OLCuOMB isolates. To our knowledge, this is the first identification of a mixed infection of bipartite and monopartite begomoviruses associated with DNA-satellites from tomato and muskmelon in Saudi Arabia. The begomovirus variants reported in this study were clustered with Iranian isolates of respective begomovirus components in the phylogenetic dendrogram. Thus, the Iranian agroecological route can be a possible introduction of these begomoviruses and/or their associated DNA-satellites into Saudi Arabia.

## 1. Introduction

Begomoviruses of the genus *Begomovirus* (family *Geminivididae*) represent a group of plant viruses infecting a wide range of dicotyledonous plants in the New World (NW) and the Old World (OW) simultaneously [1]. The virions of begomoviruses are exclusively characterized as small genomic components (~2.8–5.3 kb) encapsidated into two equally sized quasi-icosahedrons [1]. Begomoviruses are distributed across the tropical and sub-tropical to temperate agroecological regions and naturally spread through a cryptic species complex of whiteflies (*Bemisia tabaci*) [2]. Their genomes have either two equally sized components with a size of ~2.6 kb each (bipartite), i.e., DNA-A and DNA-B, or only a single component of ~2.7–2.8 kb equivalent to DNA-A of bipartite begomoviruses. The nucleotide (nt) sequence similarity of DNA-A and DNA-B of bipartite begomoviruses is limited to a short non-coding stretch of ~200 nt in the common regions (CR). This region is essential for an intact bipartite genome due to the presence of cis-acting elements for virus replication and gene expression.

The begomoviruses are phylogeographically distributed, as most of the bipartite species prevail in the NW whereas mostly monopartite species have been found in the OW often as a pathogen complex in association with DNA-satellites [3,4]. The genomic organization of monopartite begomoviruses and DNA-A of the OW bipartite begomoviruses is analogous with two virion-sense-oriented genes for movement (V2) and encapsidation (CP), four genes in the complementary-sense orientation for replication (Rep and REn), regulation of transcription (TrAP), and countering host defense (TrAP and AC4) [2]. The begomovirus genome follows a bidirectional transcription of the viral and complementary units separated by the common region of ~200 nt. The cognate DNA-A and DNA-B components share a high (>94%) sequence identity in their CRs. The DNA-satellites either have a significant impact on the disease development (betasatellites) [5] or a minimal effect on the viral infection (alphasatellites and deltasatellites) [3,6]. Noticeably, there are few exceptional examples of indigenous bipartite begomoviruses in the OW [7] and/or monopartite begomoviruses in the NW [8].

Several factors, including the global spread of the whitefly vector, the wide distribution of non-cultivated host plants, and human activities, have facilitated the emergence of new begomovirus combinations or the spread of an established begomovirus species in a unique geographical niche [9,10]. Although the indigenous begomovirus species in an area are genetically divergent from the introduced species, they may undergo a parallel or local evolution [11]. Some of the recent examples include the spread of tomato yellow leaf curl virus (TYLCV) from the OW into the NW during the 1990s [12], the spread of NW squash leaf curl virus into the Middle East [13], and the spread of cotton leaf curl disease (CLCuD) from the Indo–Pak subcontinent into China [14]. A most recent example is the cross-continental spread of OW watermelon chlorotic stunt virus (WmCSV) into Mexico and the USA [15,16]. The discovery of tomato leaf curl Palampur virus (ToLCPMV) in cucurbits, tomato, and melon crops in Iran is another instance of long-distance begomovirus spread [17]. The ToLCPMV epidemic has been devastating for cucurbit production in Iran, and some cucumber-grown protected farms reported huge economic losses [18].

Many monopartite and bipartite begomoviruses have been reported from the Arabian Peninsula, infecting various crops. Among the begomoviruses reported from Saudi Arabia, tomato yellow leaf curl virus (TYLCV) and tomato leaf curl Sudan virus (ToLCSDV) are two important begomoviruses infecting the tomato (*Solanum lycopersicum* L.) crop [19,20,21]. Similarly, cotton leaf curl Gezira virus (CLCuGeV) has been reported from okra plants in Saudi Arabia [19]. Moreover, symptomatic tomato and muskmelon (*Cucumis melo* L.) plants were identified during a field survey in the Al Ahsa region of Saudi Arabia, and preliminary screening confirmed the presence of begomovirus infection in those plants. Despite the vast scope of research in this area, the conventional contemporary approaches to detecting and characterizing the plant virome provide limited information on predominant viral genomes [19,22]. The high specificity of the primers and the detection of the most abundant viral genomes are the two major hindrances to a true assessment of the genetic diversity of begomoviruses and the associated DNA-satellites from the infected host plant via conventional methods. In Saudi Arabia, tomatoes and cucurbits are cultivated on small farms under natural or protected farming systems, and a significant share goes to local consumption [23]. Two important tomato-infecting begomoviruses in Saudi Arabia are TYLCV and tomato leaf curl Sudan virus (ToLCSDV) [21]. Keeping in view the importance of tomato and muskmelon crops, a detailed study was designed to investigate the suspected begomovirus infection from these crops on a larger scale. This study found a mixed infection of the CLCuGeV complex along with TYLCV and ToLCPMV in tomato and muskmelon plant samples. All the begomovirus isolates were closely related to their respective isolates reported from Iran.

## 2. Results

### 2.1. Illumina High-Throughput Data Analysis

**Figure 1:**
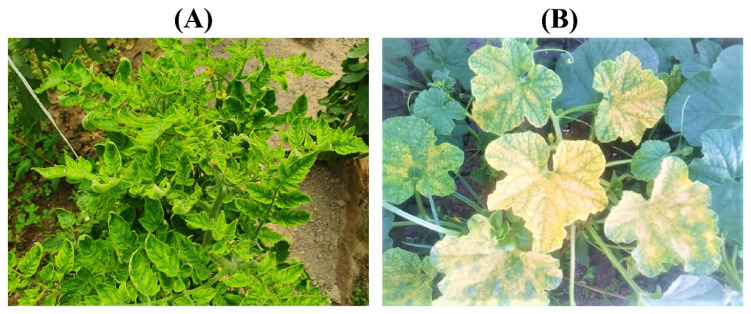
Virus infection symptoms on plants from Al-Ahsa region, Saudi Arabia; (**A**) symptomatic tomato plants showing leaf curling and yellowing; and (**B**) muskmelon plants showing leaf yellowing and mosaic symptoms.

Tomato and muskmelon plants with typical begomovirus symptoms were observed in the four open field plots of Al-Hufuf and Qateef municipalities in the Al-Ahsa province, Saudi Arabia (Figure 1). Nearly ~20–30% of the plants in each field were observed as symptomatic, showing leaf curling or yellowing symptoms. The preliminary analysis based upon core CP amplification of begomoviruses showed that all symptomatic tomato plants from Al-Hufuf and Qateef were positive. However, in muskmelon plants, only two samples from Qateef were positive for the presence of begomovirus infection. The sequenced core CP amplicons showed their highest identities to TYLCV sequences in the positive tomato and muskmelon plants. Based upon the initial detection, the tomato samples 5ToH-N and 5ToH-S were obtained from Al-Hufuf and 1ToQ-N and 1ToQ-S from Qateef, while two muskmelon samples 18MeQ-N and 18MeQ-S were selected from Qateef for Illumunia high-throughput sequencing. The Illumina sequencing produced six DNA libraries yielding 2,339,052, 2,169,526, 2,585,212, 2,792,606, 2,885,258, and 2,442,629 raw paired reads from 5ToH-N, 5ToH-S, 1ToQ-N, 1ToQ-S, 18MeQ-N, and 18MeQ-S samples, respectively. The assembled begomovirus contigs were subjected to a BLASTn search in the NCBI GenBank database. All six samples were evaluated for the presence of putative bipartite and monopartite begomovirus genomes together with DNA-satellites. Two sequences, 5THT_N and 5THT_S (2757 nt), were identified from the tomato samples 5ToH-N and 5ToH-S from the Al-Ahsa region. Similarly, the sequences 1TQT_N (2764 nt), 1TQG_N (2763 nt), 1TQa_N (1467 nt), and 1TQb_N (1356 nt) were identified from the tomato sample 1ToQ-N, while the sequences 1TQT_S (2764 nt), 1TQG_S (2763 nt), 1TQa_S (1466 nt), and 1TQb_S (1327 nt) were identified from the sample 1ToQ-S. Nevertheless, sequences 18MQG_N (2765 nt), 18MQP_N (2756 nt), 18MQB_N (2720 nt), 18MQa_N (1467 nt), and 18MQb_N (1356 nt) were identified from the sample 18MeQ-N. The sequences 18MQG_S (2765 nt), 18MQP_S (2756 nt), 18MQB_S (2720 nt), 18MQa_S (1467 nt), and 18MQb_S (1356 nt) were identified from the sample 18MeQ-S (Table 1).

### 2.2. Sequence Comparisons and Identification of Begomoviruses and DNA-Satellites

**Figure 2:**
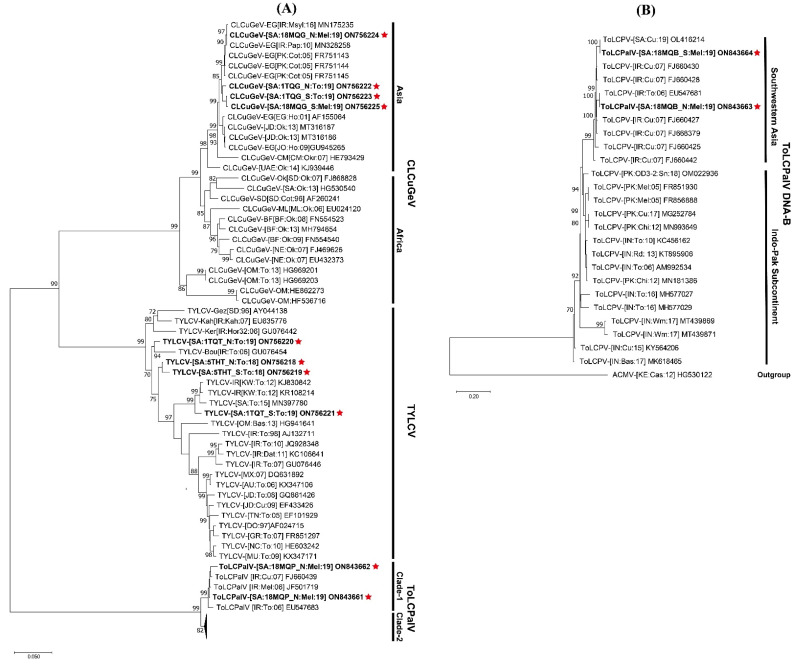
Maximum likelihood phylogenic dendrograms showing the evolutionary relatedness of (**A**) full-length genomes of identified cotton leaf curl Gezira virus (CLCuGeV), tomato yellow leaf curl virus (TYLCV), tomato leaf curl Palampur virus (ToLCPalV) DNA-A, and (**B**) ToLCPalV DNA-B. In each case, the full-length sequences of the most closely related begomvovirus genomes were retrieved from the NCBI GenBank database for comparison with the identified respective begomovirus genomes. The host plant species, geographical origins, and database accession numbers of each isolate used in this tree have been included. The begomovirus isolates identified in this study are written in bold text and indicated with a red star. The trees were constructed with percentage bootstrap values set at 1000 replicates. For taxonomic abbreviations of begomovirus species, the guidelines by Zerbini et al. [1] were followed. The ToLCPalV isolates reported from the Indo–Pak subcontinent were merged. Abbreviations used for begomovirus species were cotton leaf curl Gezira virus (CLCuGeV), tomato yellow leaf curl virus (TYLCV), tomato leaf curl Palampur virus (ToLCPalV), and African cassava mosaic virus (ACMV).

The SDT-based pairwise nt sequence identities of the identified sequences showed that tomato and muskmelon samples were infected with begomoviruses alone or in association with DNA-satellites. The sequences 5THT_N, 5THT_S, 1TQT_N, and 1TQT_S from tomato plants were 95.7–100% identical to each other. The sequence 1TQT_N shared its highest nt sequence identity of 96.7% with a TYLCV isolate (GU076454) of the “Boushehr” strain infecting tomatoes in Iran [24]. Other sequences shared their highest nt sequence identities of 95.3–97.2% with the TYLCV isolate (KR108214) of the “Iran” strain infecting cucumbers in Kuwait [25]. In the phylogenetic dendrogram, all these isolates shared the same well-supported clades with other TYLCV isolates of the “Boushehr” and “Iran” strains (Figure 2A). The sequences 1TQG_N, 1TQG_S, 18MQG_N, and 18MQG_S were 98.3–99.7% identical to each other, sharing their maximum nt sequence identities between 98.7% and 100% with the CLCuGeV isolate (MN328258) of the “Egypt” strain reported from papaya crops in Iran [26]. Meanwhile, these sequences also shared high-nt sequence identities with CLCuGeV isolates reported from cotton in Pakistan [27]. In the phylogenetic dendrogram, these isolates were grouped in the same clade as other CLCuGeV isolates of the “Egypt” strain reported from Iran and Pakistan (Figure 2A). The sequences 18MQP_N and 18MQP_S from muskmelon shared 98.9% mutual nt sequence identity, and the highest nt sequence identities were 99–99.6% with ToLCPalV DNA-A isolate (FJ660439) identified from cucumber in Iran [17]. Meanwhile, the mutual nt sequence identity of 18MQB_N and 18MQB_S was 96.3%. The sequence 18MQB_N shared its highest nt sequence identity of 100% with ToLCPalV DNA-B isolate (EU547681), and 18MQB_S shared nt sequence identity (97.9%) with ToLCPalV DNA-B isolate (FJ660430) reported from tomato in Iran, respectively [17]. In the phylogenetic dendrograms, the identified ToLCPalV DNA-A and DNA-B isolates grouped into well-supported clades with other isolates reported from cucumber and tomato crops in Iran (Figure 2A,B). The pairwise nt sequence comparison, phylogenetic analysis, and following the species demarcation of the begomovirus study group in the International Committee for the Taxonomy of Viruses (ICTV) identified the isolates 5THT_N, 5THT_S, 1TQT_N, and 1TQT_S as TYLCV variants from tomato. Furthermore, the isolates 1TQG_N, 1TQG_S, 18MQG_N, and 18MQG_S are members of CLCuGeV, whereas the isolates 18MQP_N and 18MQP_S are members of ToLCPalV DNA-A. Similarly, the isolates 18MQB_N and 18MQB_S represent DNA-B of ToLCPalV.

**Figure 3:**
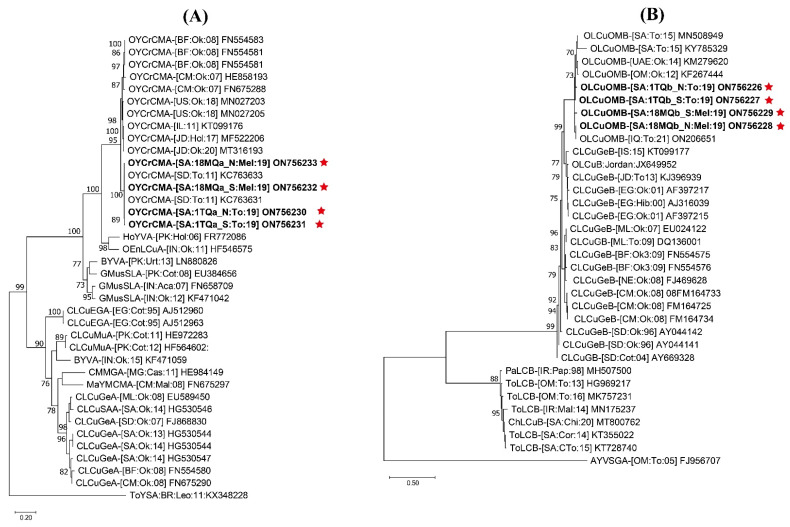
Maximum likelihood phylogenic dendrograms showing the evolutionary relatedness of (**A**) full-length sequences of identified okra yellow crinkle Cameroon alphasatellite (OYCrCMA) and (**B**) okra leaf curl Oman betasatellite (OLCuOMB). In each case, the full-length sequences of the most closely related DNA-satellite sequences were retrieved from the NCBI GenBank database for comparison with the identified respective DNA-satellite. The host plant species, geographical origins, and database accession numbers of each isolate used in this tree have been provided. The DNA-satellite isolates identified in this study are written in bold text and indicated with a red star. The trees were constructed with percentage bootstrap values set at 1000 replicates. The guidelines suggested by Briddon et al. [6] and Briddon et al. [31] were followed for taxonomic abbreviations of alphasatellite and betasatellites. Abbreviations used for alphasatellites were; Ageratum yellow vein Singapore alphasatellite (AYVSGA), bhendi yellow vein alphasatellite (BYVA), Cassava mosaic Madagascar alphasatellite (CMMGA), cotton leaf curl Egypt alphasatellite (CLCuEGA), cotton leaf curl Gezira alphasatellite (CLCuGeA), cotton leaf curl Multan alphasatellite (CLCuMuA), cotton leaf curl Saudi Arabia alphasatellite (CLCuSAA), Gossypium mustelinum symptomless alphasatellite (GMusSLA), Hollyhock yellow vein alphasatellite (HoYVA), Malvastrum yellow mosaic Cameroon alphasatellite (MaYMCMA), okra enation leaf curl alphasatellite (OEnLCuA), okra yellow crinkle Cameroon alphasatellite (OYCrCMA), and tomato yellow spot alphasatellite (ToYSA). Abbreviations used for betasatellite species were chili leaf curl betasatellite (ChLCuB), cotton leaf curl Gezira betasatellite (CLCuGeB), okra leaf curl Oman betasatellite (OLCuOMB), papaya leaf curl betasatellite (PaLCB), and tomato leaf curl betasatellite (ToLCB).

The sequences 1TQa_N, 1TQa_S, 18MQa_N, and 18MQa_S were 99.2–99.8% identical to each other and shared their nt sequence identities at 99.5–100% with an okra yellow crinkle Cameroon alphasatellite (OYCrCMA) isolate (KC763633) reported from a tomato crop in Sudan [6,28]. In the phylogenetic dendrogram, these isolates were grouped into a separate clade along with other OYCrCMA isolates reported from tomatoes in Sudan (Figure 3A). Based on the taxonomic criteria for the family Alphasatellitidae [6,29], the sequences 1TQa_N, 1TQa_S, 18MQa_N, and 18MQa_S are members of OYCrCMA infecting tomato and muskmelon in Saudi Arabia.

The sequences 1TQb_N, 1TQb_S, 18MQb_N, and 18MQb_S shared 93.4–98.3% mutual nt sequence identity, whereas 1TQb_S, 18MQb_N, and 18MQb_S shared the highest nt sequence identities at 95.6–99.7% with an okra leaf curl Oman betasatellite (OLCuOMB) isolate (ON206651) reported from the tomato crop in Iraq. However, the sequence 1TQb_N shared its highest nt sequence identity of 95.9% with an OLCuOMB isolate (KF267444) reported from the okra crop in Oman [30]. The phylogenetic analysis grouped these isolates with other OLCuOMB isolates reported from the Arabian Peninsula (Figure 3B). According to the taxonomic guidelines for the genus *Betasatellite* [31], the sequences 1TQb_N, 1TQb_S, 18MQb_N, and 18MQb_S are variants of OCuOMB in Saudi Arabia.

The presence of all identified begomovirus and DNA-satellite components in the respective plant samples was confirmed using specific primer sequences for each component.

### 2.3. Identification of Putative Recombination Events

**Figure 4:**
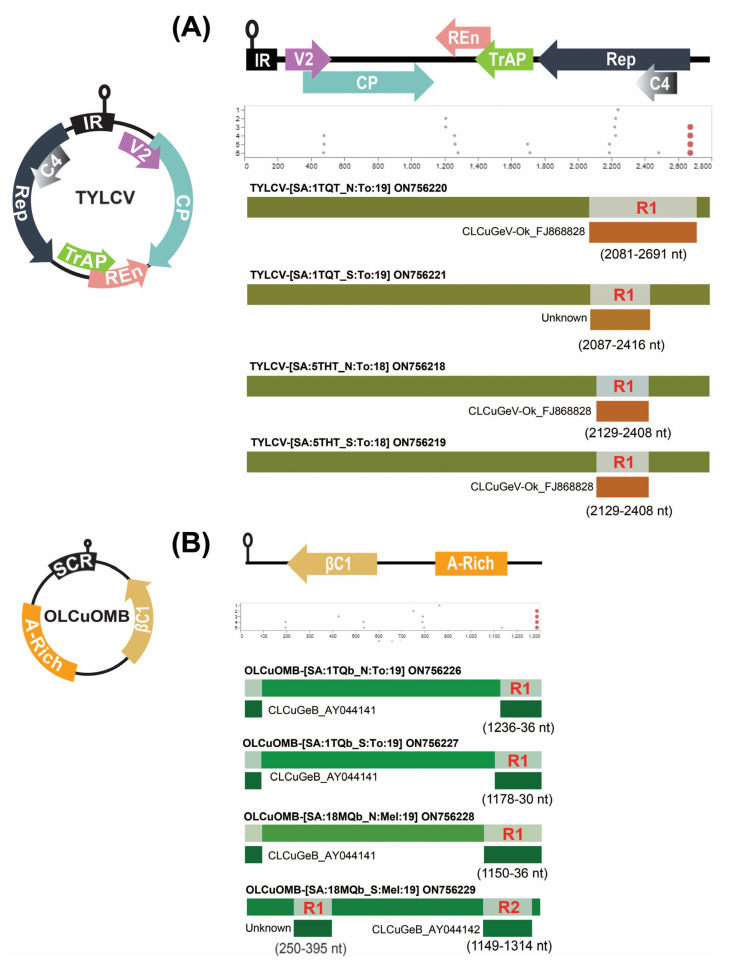
Detection of recombination events using Recombination Detection Program (RDP5.0). (**A**) Recombination analysis of the potential recombinant begomovirus and (**B**) betasatellite isolates. The graphical representation of begomovirus and betasatellite genomes is followed by GARD and RDP analyses, respectively.

Initially, the automated GARD tool available in Datamonkey was used to detect any recombination signals within the genomic regions of the identified begomovirus or DNA-satellite genomes. The GARD analysis found strong and weak recombination signals within DNA-A and betasatellite genomic regions (Figure 4). The data obtained from RDP analysis revealed that potential recombination breakpoints were only detected in the TYLCV isolates 5THT_N, 5THT_S, 1TQT_N, and 1TQT_S (Table 2). Potentially, the TYLCV isolate from Iran (KR108214) and the CLCuGeV isolate from Sudan (FJ868828) were identified as major and minor parents (Figure 4A; Table 2). Whereas, for 1TQT_S, the TYLCV isolate 1TQT_N and the CLCuGeV isolate 18MGQ_S (identified in this study) were found to have major and minor parental sequences, respectively. No significant recombination events could be detected in the CLCuGeV and ToLCPalV DNA-A or DNA-B components. Similarly, the alphasatellite isolates were also found to be non-recombinants. Moreover, the OLCuOMB isolates 1TQb_N, 1TQb_S, and 18MQb_N showed one recombination event with OLCuOMB isolates from the UAE (KM279620) and a CLCuGeB isolate from Sudan (AY044141) as the major and minor parents, respectively (Figure 4B; Table 2). While the OLCuOMB isolate 18MQb_S showed two recombination breakpoints at the nt position 250–395 with OLCuOMB from Saudi Arabia (KY785329) as a major parent and an unknown minor parent. The second recombination breakpoint was found at the nt coordinates 1149–1314 with an OLCuOMB isolate from the UAE (KM279620) as a major parent and a CLCuGeB isolate from Sudan (AY044142) as a minor parent, respectively (Figure 4B; Table 2).

## 3. Discussion

In Saudi Arabia, tomatoes and cucurbits are cultivated on small farms under natural or greenhouse farming systems [23]. Two important begomoviruses, TYLCV and tomato leaf curl Sudan virus (ToLCSDV), are known to affect tomato production in Saudi Arabia [21]. Only a limited number of studies are available on the molecular characterization of begomoviruses in Saudi Arabia; thus, information on economically important geminiviruses is scarce [21,32]. Using a high-throughput sequencing approach, we found complete genomes of both monopartite and bipartite begomoviruses from field-infected tomato and muskmelon crop plants in Saudi Arabia. We also detected an exclusive monopartite begomovirus complex as a mixed infection. Our results of nt sequence identities, phylogenetic inferences, and recombination analysis suggest that the TYLCV isolates were likely of Iranian origin. The phylogenetic inferences also showed that the “Boushehr” and “Iran” strains of TYLCV might have been introduced from Iran and Kuwait into Saudi Arabia on two different occasions. A recombination event (supported by seven algorithms) in all TYLCV isolates with a TYLCV isolate from Kuwait (KR108214) as the major parent further supports this hypothesis. Iran has been considered a possible center of TYLCV diversification due to the presence of at least five TYLCV strains in the country [33]. The CLCuGeV isolates identified from tomato and muskmelon represented members of the “Egypt” strain of CLCuGeV and showed very high nt sequence identities with a CLCuGeV isolate recently deposited from Iran [26] and another isolate reported from Pakistan [27]. Interestingly, the CLCuGeV isolates from Saudi Arabia were separately clustered with the CLCuGeV isolates reported from Asia and more closely grouped with the CLCuGeV isolates from Pakistan and Iran. CLCuGeV is an African *Malvaceae*-adapted monopartite begomovirus, and now it has spread into the Indo–Pak subcontinent, the Middle East [19,34,35], and has recently been identified in the USA [36]. In Saudi Arabia, CLCuGeV has been reported earlier from okra [19]; however, the identification of CLCuGeV isolates from new crop hosts is alarming and may lead to widespread crop infection.

The ToLCPalV isolates also showed very high nt sequence identity with ToLCPalV isolates reported from Iran [17]. ToLCPalV is a bipartite begomovirus widely spread in India and Pakistan, where it has been reported to infect many host plants, including tomato and cucurbits [37,38,39,40,41], and recently a weed [42]. Later, it was spread into Iran in 2009 [43], and until now, it has been reported from common bean, cucumber, melon, pumpkin, and watermelon crops in Iran [17,18,43,44]. The introduction of ToLCPalV in Saudi Arabia indicates its widespread occurrence in the new agroecological regions. Similarly, the dissemination of two different Iranian TYLCV strains into Saudi Arabia has likely occurred through the transportation of virus-infected vegetative sources, or it might be the cross-border dissemination of viruliferous whiteflies from Iran. Investigating the mode of virus transmission is beyond the scope of this study; however, evidence of seed transmission of tomato infecting begomoviruses is well reported [45,46]. The introduction of diverse begomovirus species in the Saudi Arabian agroecosystem with foreign origin, points to an alarming situation for agriculture in Saudi Arabia and the Middle East. It would be difficult to answer this overland introduction of OW begomoviruses into Saudi Arabia. However, probable speculation could be the intensive trade, cross-border traveling of sizeable expatriates, and transportation means as compared to the long-distance dispersal of viruliferous arthropod insects due to the presence of the vast Arabian Desert as a major obstacle. However, it cannot be completely ruled out because all begomovirus isolates identified in this study were closely clustered with the isolates from Iran. Historically, Iran is one of the pioneer regions where agriculture has a long history and tradition [47]. Moreover, Iran has been ranked fifth in terms of global agroclimatic diversity [48], and thus, it is the major vegetable producer in the Middle East and mid-Eurasia [33]. Due to diverse climatic conditions, Iranian agroecology provides conducive conditions for a probable virus outbreak and whitefly proliferations Nevertheless, the history of agriculture, diverse host plants, high genetic diversity of different begomovirus species, and *B. tabaci* biotypes indicate that at least some begomovirus species may have Iranian origin [49].

The association of OYCrCMA and OLCuOMB with CLCuGeV from tomato and muskmelon plants is the first identification of this combination from Saudi Arabia. The interaction between monopartite begomoviruses and their cognate or non-cognate betasatellites promotes virus infection. In mixed infections, many begomoviruses co-existing in natural host plants have shown neutral synergistic interactions [50,51,52], which may lead to more severe disease complexes. The finding that tomato and muskmelon plants harbor a mixed infection of CLCuGeV and its associated DNA-satellites together with TYLCV or ToLCPalV might suggest that these viruses may complement each other, as is the case of previous reports on TYLCV [50,53,54]. Nevertheless, the association of OLCuOMB and OYCrCMA might be a host-switching strategy because CLCuGeV is mostly associated with CLCuGeB and/or CLCuGeA [19,34,55]. However, this possibility requires a detailed empirical study to explain our speculation.

Furthermore, we found mixed infections of TYLCV and ToLCPalV with CLCuGeV isolates in two plants. The presence of dual or even triple infection of begomoviruses within the same plants has been consistently reported earlier [56,57,58]. The multiple begomovirus infections may cause the emergence of novel recombinants or pseudo-recombinants, which, coupled with a large vector population, can further intensify the genetic flow among begomoviruses in a particular region [59]. Geminiviruses follow a rolling circle replication mechanism; for that, the Rep protein has to bind to a specific Rep-binding iterated sequence. Thus, in bipartite begomoviruses the DNA-A and DNA-B components shared similar iteron sequences to commence successful replication [60]. This could be a reason that in mixed infections between monopartite and bipartite begomoviruses, the DNA-B component cannot be well trans-replicated by the monopartite begomovirus genome [59]. Monopartite begomovirus associated DNA-satellites either harbor their Rep protein (alphasatellites), or are flexible in their trans-replication by the helper begomovirus, i.e., betasatellites [61,62,63].

## 4. Materials and Methods

### 4.1. Plant Samples Collection, DNA Extraction and Detection of Begomovirus Genomes

Newly emerged leaf samples were collected from ten symptomatic tomato and five muskmelon plants from four field plots in Al-Hufuf and Qateef region. All samples were stored in liquid nitrogen and later stored at −80 °C until further use. Total genomic DNA was extracted using DNeasy^®^ Plant Mini Kit (Qiagen, Germantown, MD, USA). Conventional polymerase chain reaction (PCR) was performed using universal degenerate primers AC1048/AV494, amplifying ~550 bp core coat protein (CP) region of begomovirus genome [64]. After purification with the GeneJet PCR purification kit (ThermoFisher Scientific, Waltham, MA, USA), the amplified PCR products were directly sequenced at Macrogen Korea using the Sanger sequencing platform.

### 4.2. Rolling Circle Amplification (RCA) and Next Generation Sequencing

Following the preliminary analysis using universal degenerate primers for begomovirus detection, leaf samples from four tomato and two muskmelon plants were subjected to RCA with Φ-29 DNA polymerase using Illustra Templiphi amplification kit (GE Healthcare, Chicago, IL, USA). The RCA products were purified and directly sent to perform begomovirus whole genome de novo sequencing. However, the identification of any RNA viruses in the samples is hardly possible following this NGS workflow. The sequencing data were generated as Nextera XT library using Illumina MiSeq 300 bp PE platform available at Macrogen, Korea.

### 4.3. Sequence Analysis of the NGS Data and Virus Genome Assembly

Raw FASTQ-sequenced reads were first assessed for quality using FastQC (v0.11.8) [65]. The reads were then passed through Trimmomatic tool v0.39 for quality trimming [66] and adapter sequence removal with the following parameters (ILLUMINACLIP: trimmomatic_adapter.fa:2:30:10 TRAILING:3 LEADING:3 SLIDINGWINDOW:4:15 MINLEN:76). Following the quality trimming, the reads were assessed again using FastQC. Post quality check, the reads were aligned to the reference genome of the corresponding virus, i.e., tomato yellow leaf curl virus (GU076454), tomato leaf curl Palampur virus DNA-A (EU547683) and DNA-B (EU547681), cotton leaf curl Gezira virus (MN328258), cotton leaf curl Gezira alphasatellite (KC763634), and cotton leaf curl Gezira betasatellite (ON206651) with BWA-MEM2 v2.2.1 using default parameters with −k 10 T 12 flags for all samples [67]. The resulting SAM alignments were then converted to BAM format and coordinate sorted using SAM tools v1.9 [68]. The sorted alignment files were then passed through Picard Tools pipeline (http://broadinstitute.github.io/picard/, accessed on 19 September 2022) to assign all reads to the new read group in the output BAM file. Finally, the consensus sequences were assembled with Samtools’ mpileup function and piping the output to iVar consensus v1.3 [69]. Briefly, the Samtools pileup command generated a pileup of variants from the bam files with parameters including orphan read pairs (−A) and a minimum base quality for mapping set to 0 (−Q 0). The iVar consensus command was run with the default parameters except that the minimum depth to call a consensus was set to 20 (−m 20).

### 4.4. PCR-Based Confirmation of Begomovirus Genomic Components

The RCA products from the tomato and muskmelon plant samples were diluted (10×) and employed as a template to re-confirm the presence of each begomovirus and DNA-satellite component through PCR reactions using specific primers (Table 3). The PCR amplicons were purified using GeneJet Gel extraction kit (ThermoFisher Scientific, Waltham, MA, USA) and were sequenced through the Sanger sequencing platform (Macrogen, Seoul, Korea). The obtained sequences were compared non-redundantly using NCBI GenBank database to validate their identity.

### 4.5. Determination of Pairwise Nucleotide Sequence Identities

Initially, the BLASTn tool in the NCBI GenBank database was used to estimate the nt sequence identities of the genomic components. The highest BLASTn hits were retrieved from the database and were used to perform pairwise nt sequence identities of each genomic component individually. The nt sequences of the full-length components were aligned in MEGA-11 software using ClustalW algorithm [70]. The suggested Species Demarcation Tool (SDTv1.2) was used to estimate the pairwise nt sequence identities following the guidelines for geminiviruses demarcation [71]. The individual open reading frames (ORFs) and non-translated regions (NTRs) were also compared in the publicly available NCBI ORF finder tool (https://www.ncbi.nlm.nih.gov/orffinder/, accessed on 12 June 2022).

### 4.6. Evolutionary Relatedness through Phylogenetic Dendrograms

The evolutionary relationship of each genomic component was inferred through phylogenetic dendrograms generated in the MEGA11 software. The maximum likelihood statistical method was used to compute the evolutionary distances of each dataset, while the best-fit gamma distribution with invariant sites (G + I) model was used to determine the rate of variation among each site. The phylogenetic trees were exported in EMF format and graphically simulated in Adobe Illustrator (CC) software.

### 4.7. Estimation of Recombination Breakpoints

A primary dataset including 300 full-length begomovirus DNA-A, 240 full-length DNA-B, and 200 DNA-satellite sequences was retrieved and assembled with the respective genomic components in this study using MEGA11 software. The complete assembly of the aligned DNA-A and DNA-B sequences was exported in FASTA format to be used for recombination analysis. The putative recombination events were inferred using GARD and recombination detection program (RDP v5.0) [72]. Seven different algorithms were selected for RDP5, and only those recombination events and breakpoints were considered that were supported by at least three different algorithms. Default settings were used for recombination analysis, and a cut-off value of 0.05 was selected as a Bonferroni-corrected *p*-value.

## 5. Conclusions

In conclusion, we revealed that the begomoviruses and associated DNA-satellite isolates identified in this study are members of begomovirus species from the OW. Such mixed infections of multiple bipartite and monopartite begomoviruses associated with DNA-satellites have not been reported earlier from tomato and muskmelon plants in Saudi Arabia. It was found that the begomovirus isolates were closely grouped with the isolates reported from Iran. Iran has one of the ancient agricultures and its diverse agroecological environment makes it a hot spot with rich genetic diversity of begomoviruses and whitefly. Thus, it might be possible that these begomoviruses have been prevailing in the agroecological regions of Iran for a long period and are now spreading to the neighboring regions either via viruliferous insects or via transportation of the infected plant material. This information may help to understand the begomovirus etiology in this region and may pave the way toward better disease management strategies. The identification of complete genomic components from this study may help to explain the evolutionary dynamics of begomoviruses in Saudi Arabia. Further crops monitoring and empirical studies are required to ascertain precisely the introduction and spread of begomoviruses from Southeast Asia and Africa into the agroecological regions of Saudi Arabia. Strict quarantine measures should be followed to control the cross-border international trade of agricultural products and infected plant materials (ornamental plants in particular) to prevent the introduction of new plant virus species in this region in the future.

## Figures and Tables

**Table 1:** Sample collection and complete genome sequencing using Illumina MiSeq and Sanger sequencing platforms.

Plant	Sample	Place	TYLCV	CLCuGeV	OLCuOMB	OYCrCMA	ToLCPalV DNA-A	ToLCPalV DNA-B
Tomato	5ToH-N	Al-Hufuf	5THT_N (ON756218) (95.3)	-	-	-	-	-
	5ToH-S		5THT_S (ON756219) (96.4)	-	-	-	-	-
	1ToQ-N	Qateef	1TQT_N (ON756220) (96.7)	1TQG_N (ON756222) (98.7)	1TQb_N (ON756226) (95.9)	1TQa_N (ON756230) (99.5)	-	-
	1ToQ-S		1TQT_S (ON756221) (97.2)	1TQG_S (ON756223) (98.7)	1TQb_S (ON756227) (96.8)	1TQa_S (ON756231) (99.5)	-	-
Melon	18MeQ-N		-	18MQG_N (ON756224) (100)	18MQb_N (ON756228) (99.7)	18MQa_N (ON756232) (100)	18MQP_N (ON843661) (99.6)	18MQB_N (ON843663) (100)
	18MeQ-S		-	18MQG_S (ON756225) (98.6)	18MQb_S (ON756229) (95.6)	18MQa_S (ON756233) (99.5)	18MQP_S (ON843662) (99.0)	18MQB_S (ON843664) (97.9)

The numbers in the parenthesis under each isolate represent the highest percentage (%) nucleotide sequence identity of that isolate with the respective species of begomovirus or DNA-satellites.

**Table 2:** Recombination detection using seven different methods in RDP5.

Event No.	Recombinant	Breakpoints *		Parents **		Methods ***	*p*-Value
		Begin	End	Major	Minor		
1.	TYLCV_1TQT_N (ON756218)	2081	2691	TYLCV_KR108214 (93.4)	CLCuGeV_FJ868828 (79.0)	***R***,***G***,***B***,***M***,***C***,***S***,***3S***	4.56 × 10^−41^
2.	TYLCV_1TQT_S (ON756219)	2087	2416	TYLCV_1TQT_N (95.8)	Unknown (CLCuGeV_18MGQ_S) (74.4)	***R***,***G***,***B***,***M***,***C***,***S***,***3S***	5.63 × 10^−53^
3.	TYLCV_5THT_N (ON756220)	2129	2408	TYLCV_KR108214 (95.3)	CLCuGeV_FJ868828 (78.6)	***R***,***G***,***B***,***M***,***C***,***S***,***3S***	4.56 × 10^−41^
4.	TYLCV_5THT_S (ON756221)	2129	2408	TYLCV_KR108214 (96.4)	CLCuGeV_FJ868828 (77.1)	***R***,***G***,***B***,***M***,***C***,***S***,***3S***	4.56 × 10^−41^
5.	OLCuOMB_1TQb_N (ON756226)	1234	36	OLCuOMB_KM279620 (95.5)	CLCuGeB_AY044141 (89.6)	R,G,***M***,***S***,***3S***	6.75 × 10^−29^
6.	OLCuOMB_1TQb_S (ON756227)	1178	30	OLCuOMB_KM279620 (96.1)	CLCuGeB_AY044141 (90.7)	R,G,***M***,***S***,***3S***	6.75 × 10^−29^
7.	OLCuOMB_18MQb_N (ON756228)	1150	36	OLCuOMB_KM279620 (94.8)	CLCuGeB_AY044141 (90.3)	R,G,***M***,***S***,***3S***	6.75 × 10^−29^
8.	OLCuOMB_18MQb_S (ON756229)	250	395	OLCuOMB_KY785329 (87.3)	Unknown (CLCuGeB_AY044141) (88.1)	***R***,***G***,B,M,***C***,***S***,***3S***	1.66 × 10^−10^
		1149	1314	OLCuOMB_KM279620 (93.7)	CLCuGeB_AY044142 (87.8)	***G***,***M***,C,S,***3S***	9.27 × 10^−13^

3S, Sequence Triplets; B, Bootscan; C, Chimarea; G, Geneconv; M, MaxChi; R, RDP; S, SiScan. * Each breakpoint is referred to by the nucleotide position on the respective reference genome. ** The numbers in parenthesis represents the percentage nucleotide sequence identity of each recombinant isolate with the respective begomovirus or betasatellite species as major and minor parent. *** The methods showing cut-off *p*-values > 0.05 are indicated in bold and italicized text. The method showing the highest *p*-value is underlined and given in the column.

**Table 3:** Oligonucleotide sequences used in this study.

Primers	Primer Sequence	Nucleotide Position	PCR Product
AC1048	GGRTTDGARGCATGHGTACATG		Core coat protein [64]
AV494	GCCYATRTAYAGRAAGCCMAG		
TY-Cp_F	TGAAGGCCCATGTAAAGTCCAG	495–516	TYLCV
TY-Cp_R	CATAGAAATAGATACGTATTTTC	1026–1048	
CG-Cp_F	GTGTGAAGGTCCATGTAAGGTCC	525–547	CLCuGeV
CG-Cp_R	GTAGCATACACAGGATTAGAAG	1034–1055	
PA-Cp_F	AGCTCTGACGTGCCCAGGGGCT	460–481	ToLCPalV DNA-A
PA-Cp_R	CACCGAATCGTAAAAATAGATC	1020–1041	
PB-C1C_F	CGTTTGTGAGCGCGTACTCAATAC	2062–2085	ToLCPalV DNA-B
PB-C1C_R	AATATTATATACGAAAGGCCCCTT	2697–2720	
OY-RC_F	GTAATTGTAATGGCTAATTTCCTC	873–896	OYCrCMA
OY-RC_R	GGTAATACTGGAGCCGGCCTCAG	1448–03	
Ob-BC1_F	ACACTGATGATTTATTTAGTATGC	246–269	OLCuOMB
Ob-BC1_R	ATGACTATCACATTCAGGAACACC	574–597	

## Data Availability

All the data can be accessed under accession numbers ON756218-33 and ON843661-64 in the NCBI GenBank database (https://www.ncbi.nlm.nih.gov/nucleotide/, accessed on 12 June 2022).
